# Importation of Fosfomycin Resistance *fosA3* Gene to Europe

**DOI:** 10.3201/eid2202.151301

**Published:** 2016-02

**Authors:** Ana C. Mendes, Carla Rodrigues, João Pires, José Amorim, Maria Helena Ramos, Ângela Novais, Luísa Peixe

**Affiliations:** Universidade do Porto, Porto, Portugal (A.C. Mendes, C. Rodrigues, J. Pires, Â. Novais, L. Peixe);; Centro Hospitalar do Porto, Porto (A.C. Mendes, M.H. Ramos);; Botelho Moniz Análises Clínicas, Santo Tirso, Portugal (J. Amorim)

**Keywords:** fosfomycin resistance, fosA3, Escherichia coli, ST393, extended-spectrum β-lactams, ESBL, CTX-M-15, IncFII plasmid, community, urinary tract infection, Portugal, bacteria, antimicrobial resistance

**To the Editor**: The wide spread of *Enterobacteriaceae* resistant to last-resource therapeutic options, including extended-spectrum β-lactams, fluoroquinolones, and aminoglycosides, has re-ignited interest in old antimicrobial drugs, such as fosfomycin (*1*). Fosfomycin resistance rates are generally low (<10%) but substantially higher when carbapenemase producers are considered (15%–34%) (*1*–*3*). Resistance phenotypes have been more thoroughly investigated in *Escherichia coli* and linked to chromosomal mutations in the target (*murA*) or transporter (*glpT* and *uhpT*) genes or less frequently to plasmid-mediated fosfomycin resistance genes (*fosA*, *fosB*, *fosC*) encoding glutathione S-transferases that inactivate the drug (*4*). *fosA3* is the most prevalent gene variant, disseminated mainly in *E. coli* isolates from clinical and nonclinical origins (healthy persons, companion and food animals) in countries in Asia (China, South Korea, and Japan) (*2*–*6*) and only recently in a migratory bird in Europe (*7*). We investigated the occurrence and molecular features of 43 fosfomycin-resistant *Enterobacteriaceae* isolates (21 *E. coli*, 21 *Klebsiella pneumoniae*, and 1 *Morganella morganii*). These isolates were identified among 461 third-generation cephalosporin-resistant *Enterobacteriaceae* isolates from a community laboratory in northern Portugal during a 13-month period (August 2012–August 2013).

We screened for carriage of plasmidborne fosfomycin resistance genes (*fosA*, *fosA3*, *fosB*, *fosC2*) by PCR and sequencing (*2*,*5*). Chromosomal mutations in *murA*, *glpT*, and *uhpT* were investigated for 9 representative *E. coli* isolates (*8*) and 7 representative *K. pneumoniae* isolates with variable MICs to fosfomycin (>64 mg/L) by PCR and comparison of sequences with reference wild-type strains (*E. coli* ATCC25922 and *K. pneumoniae* type strain JCM1662) (*8*; this study). Fosfomycin-resistant isolates represented 9.3% (43/461) of the collection surveyed during the study period, which is in line with rates reported for clinical isolates from other countries (*2*,*3*). Bacterial identification and antimicrobial drug susceptibility testing to β-lactams and non–β-lactams were performed by automated methods and further confirmed by disk diffusion and agar dilution (for fosfomycin, MIC cutoff 32 mg/L) according to European Committee on Antimicrobial Susceptibility Testing guidelines (http://www.eucast.org). We screened *bla*_ESBL_ genes (*bla*_CTX-M_, *bla*_TEM_, *bla*_SHV_) by PCR and sequencing (*9*).

One (2.3%) of 43 *E. coli* isolates carried *fosA3*, *bla*_CTX-M-15_, and *bla*_TEM-1_ and contained mutations in GlpT (L297F, T348N, Q443E, E444Q) and UhpT (E350Q) (GenBank accession nos. KT832798 and KT832797, respectively), most of which were previously associated with fosfomycin resistance (*8*). This isolate was detected in a urine sample from a 61-year-old man who had a clinical history of chronic prostatitis and was associated with a urinary tract infection (UTI) acquired after travel to Asia (China, Philippines). *aac-6’-lb-cr*, *bla*_OXA-I_, and *rmtB* genes were negative by PCR. This isolate exhibited fosfomycin MIC >256 mg/L and was concomitantly resistant to cefotaxime, cefepime, aztreonam, ciprofloxacin, gentamicin, kanamycin, netilmycin, streptomycin, sulphonamide, tetracycline, tobramycin, and trimethoprim but not to carbapenems, amoxicillin/clavulanic acid, or cefoxitin. In other *E. coli* isolates, fosfomycin resistance phenotypes were linked to mutations in transporter proteins UhpT (8 isolates, E350Q) and GlpT (3 isolates, premature stop codons resulting in truncated proteins of 45, 134, or 442 aminoacids [GenBank accession nos. KT832799, KT832800, and KT832801, respectively]); however, no amino acid changes were detected in *K. pneumoniae* isolates. The detection of *fosA3* in a clinical *E. coli* isolate in Europe is alarming because of its association with *bla*_CTX-M-15_, which is highly disseminated in Portugal and other European Union countries (*9*), whereas fosfomycin is increasingly being used to treat UTIs caused by extended-spectrum β-lactams–producing *E. coli* (*1*).

Strain typing (identification of *E. coli* phylogroups and multilocus sequence typing; http://mlst.warwick.ac.uk/mlst/) revealed that this isolate belonged to phylogenetic group D_1_ and the sequence type 393 clone (*9*). This clone was not previously detected among *fosA3*-carrying isolates (*3*,*4*), but it is distributed worldwide (including Asia) linked to community-acquired UTI and multidrug resistance patterns (*9*).

Conjugative assays (solid/broth mating at 24°C/37°C using *E. coli* Hb101 azide and kanamycin resistant as recipient) and plasmid typing assessed by PCR-based replicon typing, IncFII typing formula (FAB), I-*Ceu*I pulsed-field gel electrophoresis, and hybridization (*5*) showed that both *fosA3* and *bla*_CTX-M-15_ were co-located in a conjugative F2:A−:B− plasmid (transconjugant MIC to fosfomycin >256 mg/L). Moreover, the genetic environment of *fosA3* was assessed by PCR mapping and sequencing (*2*,*6*), showing a composite transposon containing an insertion sequence *26* 323 bp upstream *fosA3*; the *orf1*, *orf2*, and *orf3* genes (homologous to regulatory ones in *K. pneumoniae* 342); and an insertion sequence (IS) *26* downstream (GenBank accession no. KT734860). The genetic platform (IS*26* composite transposon) and the IncFII plasmid variant (F2:A−:B−) are main vehicles for disseminating *fosA3* among clinical isolates, companion and food animals in Asian countries (*3*,*5*,*6*), or *bla*_CTX-M-15_ worldwide (*10*). Thus, epidemiologic and molecular data suggest that the detection of *fosA3* in a clinical isolate in Europe is associated with a travel-related infection acquired after international travel to Asia. The acquisition of *fosA3* by a successful clone and an efficient resistance plasmid, which might entail subsequent dissemination and alerts to the need of close monitoring of fosfomycin resistant isolates, is of particular concern.
